# Evaluation of the clinical characteristics and survival outcomes of invasive pulmonary aspergillosis patients

**DOI:** 10.3389/fmicb.2025.1587227

**Published:** 2025-05-01

**Authors:** Qiangsheng Feng, Xiaoqin Ha, Yuejuan Song

**Affiliations:** Department of Clinical Laboratory, The 940th Hospital of Joint Logistics Support Force of People's Liberation Army, Lanzhou, China

**Keywords:** IPA, clinical characteristics, survival analysis, biomarkers, mortality

## Abstract

**Background:**

Invasive pulmonary aspergillosis (IPA) is a severe infectious disease caused by *Aspergillus* spp. It is associated with high mortality, particularly in immunocompromised patients, as well as in those with COVID-19 pneumonia or critically ill individuals in intensive care units (ICUs). Accurate clinical diagnosis remains a significant challenge, often resulting in missed diagnoses.

**Methods:**

This study evaluated IPA inpatients diagnosed through mycological evidence and clinical criteria over 12 months. Inclusion criteria required at least one positive mycological result, including a positive culture from bronchoalveolar lavage fluid (BALF) or high-quality sputum, or a positive galactomannan antigen (GM) test.

**Results:**

A total of 216 patients were diagnosed with IPA, with a mortality rate of 68.5%. Hematologic malignancies were the primary underlying condition in 33.8% of cases. Voriconazole or posaconazole was used in 45% (98/216) of patients overall, but only 26% (32/121) of non-hematologic malignancy patients received these treatments. The 28-day survival rate for patients treated with Voriconazole/Posaconazole was 0.776 ± 0.038, compared to 0.421 ± 0.043 for untreated patients. Median survival was 130 days (95% CI, 35.3–224.7) for treated patients vs. 20 days (95% CI, 15.8–24.2) for untreated patients (*p* < 0.001). Biomarkers for IPA diagnosis demonstrated high diagnostic value, with area under the curve (AUC) values for GM, G, PCT, IL-6, WBC, NEU%, and D-dimer of 0.953, 0.983, 1.000, 0.999, 0.961, 0.996, and 1.000, respectively. GM levels >0.5 pg/ml had a positive predictive value of 52.9% (27/51), while positive mycological culture had a predictive value of 46.5% (33/71). Multivariable regression analysis identified several significant factors associated with in-hospital mortality: IPA (OR 7.509, 95% CI 4.227–13.339, *p* < 0.001), Voriconazole/Posaconazole treatment (OR 0.124, 95% CI 0.063–0.242, *p* < 0.001), ICU hospitalization (OR 5.280, 95% CI 1.549–18.002, *p* = 0.008), hematologic malignancy (OR 0.316, 95% CI 0.174–0.573, *p* < 0.001), and NEU% ≥87.25% (OR 3.409, 95% CI 1.455–7.990, *p* = 0.005).

**Conclusion:**

Non-hematologic malignancy patients with IPA were frequently undertreated with antifungal therapy. A comprehensive diagnostic approach using biomarkers, CT, mycological evidence is crucial. Key risk factors for mortality include lack of Voriconazole/Posaconazole treatment, IPA diagnosis, ICU admission, non-hematologic malignancies, and elevated NEU%.

## Highlights

- IPA is generally susceptible to all patients.
- Delayed diagnosis or prone to missed antifungal therapy in non-hematologic malignancy IPA patients, this is our most important finding.
- Diagnosis of IPA by biomarkers and Mycological evidence was very necessary.
- Mortality risk factors non-voriconazole/posaconazole, IPA, ICU hospitalization, non-hematologic malignancy and NEU% ≥ 87.25%.These four findings deserve to be widely applied clinically for empirical treatment and prevention of invasive candidiasis.

## 1 Introduction

Invasive pulmonary aspergillosis (IPA) is a severe infectious disease caused by *Aspergillus* spp., recognized as one of the most devastating lung infections due to its high morbidity and mortality rates (Latgé and Chamilos, 2019). At-risk populations include individuals with prolonged neutropenia, allogeneic hematopoietic stem cell transplants (HSCT), solid organ transplants (SOT), inherited or acquired immunodeficiencies, corticosteroid use, and other immunosuppressive conditions (Patterson et al., 2016). Diagnosis is typically confirmed through microscopy and culture of respiratory samples, histopathology in biopsy cases, and detection of galactomannan (GM) antigen in serum or bronchoalveolar lavage fluid (BALF) using immunoassays (Ledoux et al., 2020). Despite these methods, a significant proportion of IPA cases remain undetected, particularly in non-neutropenic, ICU, and COVID-19-associated patients who often exhibit atypical clinical and radiographic signs of infection. Given the clinical importance of IPA, there is a critical need to investigate diagnostic biomarkers, positive cultures from BALF or high-quality sputum, and clinical characteristics, prompting the present study.

## 2 Materials and methods

### 2.1 Study design

**Figure 1:**
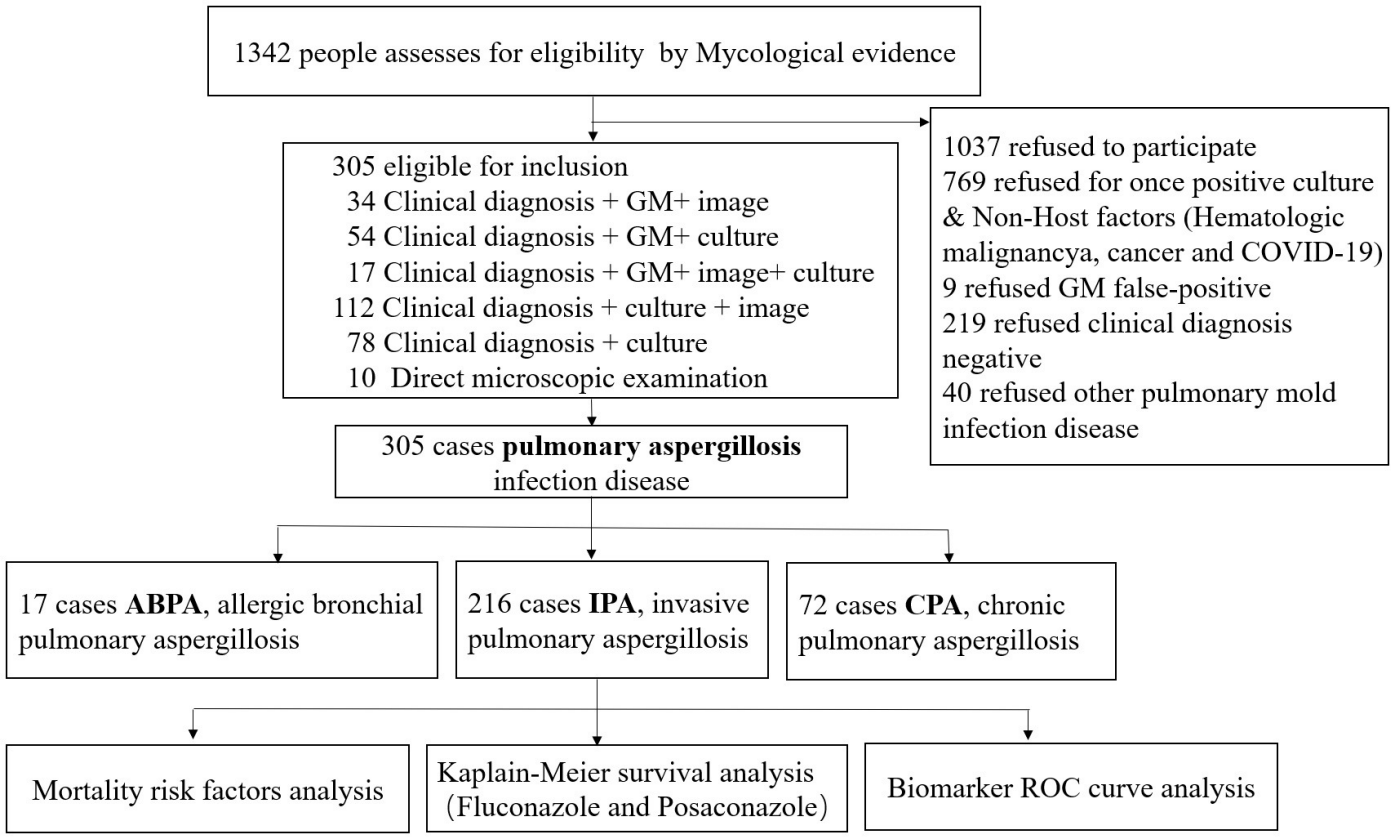
Selection criteria for the inclusion of patients with IPA (Ledoux et al., 2020).

① A total of 216 cases of IPA with pathogenic evidence were included in this study, with 57% (123/216) exhibiting at least two forms of microbiological evidence, including *Aspergillus* spp.+ GM in 21 cases, *Aspergillus* spp. culture positivity ≥2 times in 68 cases (2-8 times *Aspergillus* spp.), GM positivity ≥2 times in 18 cases, and *Aspergillus* spp. culture positive+ GM +G in 16 cases, There were 10 cases were diagnosed by direct microscopic examination (Figure 1).
② A total of 769 cases were excluded due to various reasons: single positive cultures with minimal quantitative results or suspected contamination, non-host factors such as hematologic malignancies, cancer, or COVID-19, specimen contamination or colonization, or GM false-positivity related to Piperacillin/Tazobactam use (9 cases). An additional 219 cases were excluded due to a lack of clinical diagnosis, and 40 cases were ruled out due to other pulmonary mold infections, including *Fusarium* spp., *Penicillium* spp., and mucormycosis.
③ Galactomannan antigen (GM) cut-off was detected in serum definition: single-test thresholds for positivity were defined as serum GM < 0.5 pg/mL and BALF GM < 1.0 pg/mL.

### 2.2 Setting

This study conducted a retrospective review of clinical data from patients diagnosed with IPA at the 940th Hospital of the Joint Logistics Support Force of the People's Liberation Army. Statistical analyses were performed on factors such as antifungal therapy (Voriconazole/Posaconazole), pathogens, sample types, underlying conditions, infection biomarkers, and in-hospital mortality.

### 2.3 Ethical oversight

The study was approved by the Ethics Committee of the 940th Hospital of the Joint Logistics Support Force of the People's Liberation Army (Approval No. 2023KYLL22136, December 2023). The committee waived the need for informed consent. The study adhered to the ethical standards outlined in the Declaration of Helsinki (Shephard, 1976) and its amendments.

### 2.4 Participants

This study carefully selected participants from 2018 to 2024, ultimately including 216 eligible patients who were followed for 12 months. A detailed flowchart illustrates participant eligibility and reasons for exclusion.

### 2.5 Exposure variables

Demographic and clinical characteristics were analyzed as exposure variables. These included age, sex, antifungal therapy (Voriconazole/Posaconazole), concurrent infections (COVID-19 or H1N1), ICU admission, repeated positive pathogenic evidence (≥2 instances), cancer or hematologic malignancies, imaging findings, GM value (≥0.5 pg/mL), G (≥131 pg/mL), procalcitonin (PCT), interleukin-6 (IL-6), white blood cell count (WBC), C-reactive protein (CRP), and D-dimer levels.

### 2.6 Endpoint

The primary endpoint was in-hospital mortality among IPA inpatients.

### 2.7 Biomarker analyses

Infection-related biomarkers were analyzed within 24 h of initial admission or 24 h of positive pulmonary aspergillosis culture. Specific biomarkers and associated analytical methods included: G(1-3-β-D-glucan; Jinshanchuan, Beijing, immunoturbidimetric method); Galactomannan (GM assay, Bio-Rad, USA); Procalcitonin (PCT, chemiluminescence method, Roche); C-reactive protein (CRP, immunoturbidimetry, Beckmann); Interleukin-6 (IL-6, chemiluminescence method, Roche), white blood cells (WBC count, Mindray 6900); neutrophil percentage (NEU%, Mindray 6900); D-dimer, immunoturbidimetric assay, Hiesenmikon). To avoid duplication, only one biomarker result per patient was included. For comparison, a control group of 200 non-infected patients with underlying conditions (e.g., hypertension, diabetes, chronic gastritis, gout) was included. Biomarker ranges in the control group were as follows: PCT [0.13 (0.07–0.37) ng/mL], IL-6 [22.80 (10.75–51.90) ng/mL], CRP [0.22 (0.15–0.99) mg/L], WBC [5.94 (4.76–8.14) × 10^9^/L], NEU% [67.45 (59.10–75.30)%], D-dimer [0.47 (0.18–1.30) mg/L], G [11.92 (10.0–84.8) pg/mL], GM [0.11 (0.08–0.17) pg/mL].

### 2.8 *Aspergillus* spp. culture and identification

BALF and qualified sputum specimens were inoculated onto TTC-Sabouraud agar plates and incubated at 35°C for at least 7 days. Fungal species were identified based on colony morphology and microscopic examination using lactic acid phenol Medan staining. Dentification of *Aspergillus* based on culture methods and morphology is still very important, include both macroscopic (colony features) and microscopic (structural details) observations. Macroscopic Features (Colony Characteristics): Colony Color: *Aspergillus fumigatus*: Initially white, turning blue-green with age. *Aspergillus flavus*: Yellow-green to olive-green, often with a reddish-brown reverse. *Aspergillus niger*: Initially white, becoming black due to the production of dark conidia. *Aspergillus terreus*: Cinnamon-brown to sand-colored colonies (Jorgensen and Pfaller, 2019; Long et al., 2024).

Microscopic Features: Shape: The conidial head is the most distinctive feature, consisting of a vesicle (swollen tip), phialides (spore-producing cells), and conidia (spores). Microscopic examination is essential for identifying *Aspergillus* species. Key structures to observe include Conidial HeadVesicle (Top of Conidiophore): *A. fumigatus*: Flask-shaped vesicle. *A. flavus*: Spherical vesicle. *A. niger*: Spherical vesicle. *A. terreus*: Hemispherical vesicle.

### 2.9 Statistical analyses

Statistical analyses were conducted using SPSS 22.0. Logistic regression was used to evaluate mortality risk factors, with statistical significance set at *p* < 0.05. Factors associated with in-hospital mortality were illustrated using a forest plot (*p* < 0.01). Kaplan-Meier survival curves were used to estimate survival rates, and the sensitivity and specificity of clinical biomarkers were assessed using receiver operating characteristic (ROC) curves.

## 3 Results

### 3.1 Clinical features of IPA patients

**Figure 2:**
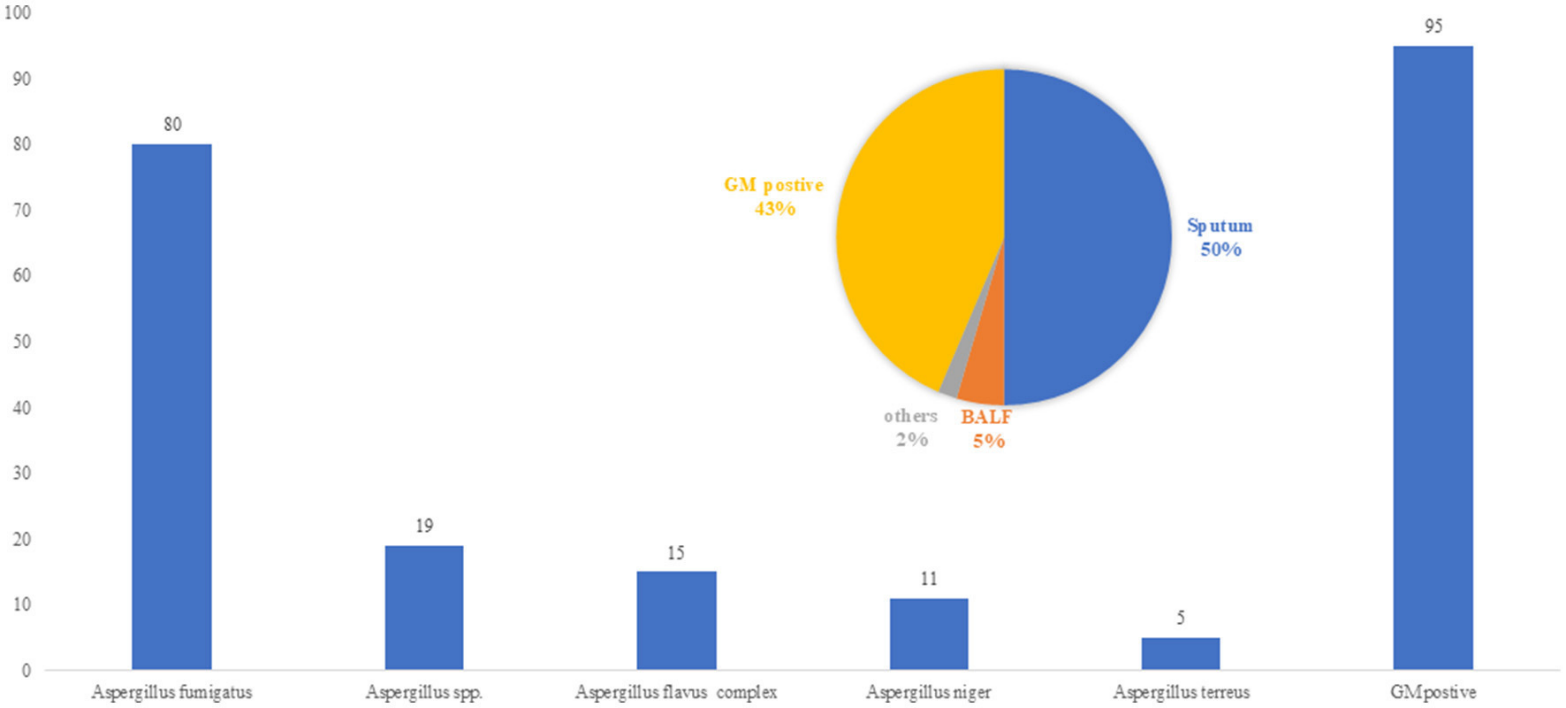
Pathogen of *Aspergillus spp*. (*n* = 216 Strains/cases). *A. fumigatus* was identified as the predominant pathogenic microorganism in IPA patients, responsible for 61.5% of cases, followed by *Aspergillus flavus* (11.5%) and *Aspergillus niger* (8.5%). Positive culture specimens included BALF, qualified sputum, and serum GM levels >0.5 pg/ml, accounting for 5%, 50%, and 43% of cases.

A total of 305 cases of pulmonary aspergillosis were diagnosed, including IPA, chronic pulmonary aspergillosis (CPA), and allergic bronchopulmonary aspergillosis (ABPA), accounting for 62.6%, 20.9%, and 4.9%, respectively. *A. fumigatus* was identified as the predominant pathogenic microorganism, responsible for 61.5% of cases, followed by *A. flavus* (11.5%) and *A. niger* (8.5%). Positive culture specimens included BALF, qualified sputum, and serum GM levels > 0.5 pg/ml, accounting for 5%, 50%, and 43% of cases, respectively, as shown in Figure 2. The primary host factors for IPA patients were hematologic malignancies (33.8%), intensive care unit (ICU) admission (18.5%), COVID-19-associated IPA (16.2%), and cancer or malignant tumors (13.9%). Additionally, there were five cases (2.3%) involving immunocompetent patients, as summarized in Table 1.

**Table 1:** Host factor in IPA patients (*n* = 216).

**Host factors**	**Cases**	**%**
Recent history of neutropenia [ < 0.5 × 109 neutrophils/L (< 500 neutrophils/mm^3^) for >10 days] temporally related to the onset of invasive fungal disease	17	7.9
Hematologic malignancy	73	33.8
Receipt of an allogeneic stem cell transplant	7	3.2
COVID-19 associated- IPA	35	16.2
Influenza virus A associated -IAPA	4	1.9
Intensive Care Unit (ICU)	40	18.5
Cancer or malignant tumor	30	13.9
Non-immunocompromised hosts	5	2.3
Calcineurin inhibitors, tumor necrosis factor-a blockers, lymphocytespecific monoclonal antibodies, immunosuppressive nucleoside analogs during the past 90 days	5	2.3

### 3.2 IPA patient survival outcomes

**Figure 3:**
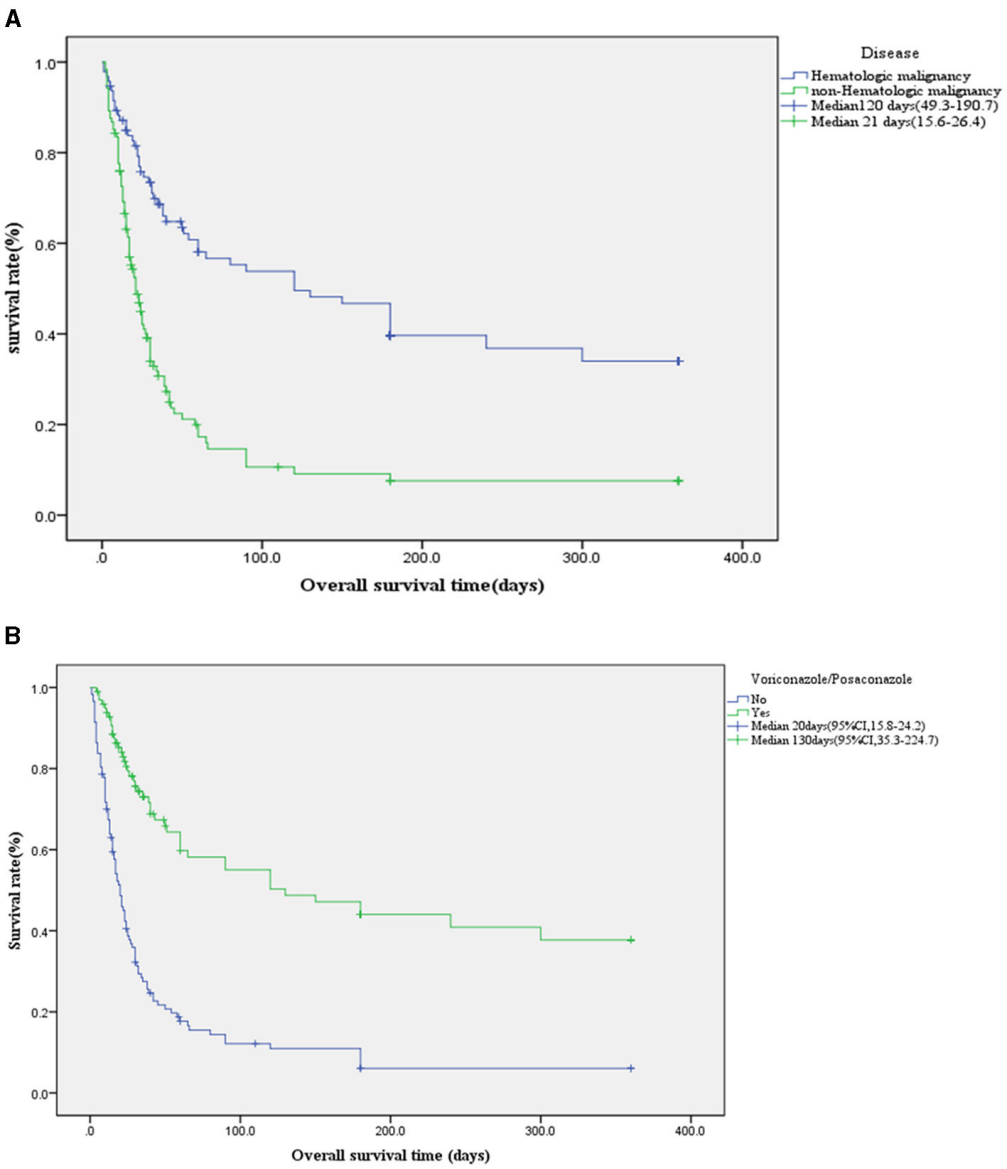
**(A)** Kaplan-Meier survival curve of underlying disease. In IPA patients with hematologic malignancies, the usage rate of voriconazole or posaconazole was 70% (66/95), and the 90-day survival rate was 0.538 ± 0.055. In contrast, for patients without hematologic malignancies, the usage rate was only 26% (32/121), with a significantly lower 90-day survival rate of 0.106 ± 0.034 (χ^2^ = 39.8, *p* < 0.001, 95% CI). **(B)** Kaplan-Meier survival curve using voriconazole or posaconazole in IPA (*n* = 216 cases). The survival rates for voriconazole or posaconazole vs. non-use at 28 and 90 were 0.769 ± 0.044 vs. 0.359 ± 0.045, 0.550 ± 0.058 vs. 0.122 ± 0.033, respectively. The median survival time was 130 days (95% CI, 35.3–224.7) for voriconazole or posaconazole users compared to 20 days (95% CI, 15.8–24.2) for non-users. Log-rank (Mantel-Cox) analysis showed χ^2^ = 53.1, *p* < 0.001, 95% CI.

Among 216 IPA patients, the mortality rate was 68.5%, Voriconazole or Posaconazole was used in 45% of patients (98/216). In IPA patients with hematologic malignancies, the usage rate of Voriconazole or Posaconazole was 70% (66/95), and the 90-day survival rate was 0.538 ± 0.055. In contrast, for patients without hematologic malignancies, the usage rate was only 26% (32/121), with a significantly lower 90-day survival rate of 0.106 ± 0.034 (χ^2^ = 39.8, *p* < 0.001, 95% CI; Figure 3A). The survival rates for Voriconazole or Posaconazole vs. non-use at 28, 42, 90, and 180 days were 0.769 ± 0.044 vs. 0.359 ± 0.045, 0.674 ± 0.052 vs. 0.227 ± 0.040, 0.550 ± 0.058 vs. 0.122 ± 0.033, and 0.440±0.059 vs. 0.061±0.025, respectively. The median survival time was 130 days (95% CI, 35.3–224.7) for Voriconazole or Posaconazole users compared to 20 days (95% CI, 15.8–24.2) for non-users. Log-rank (Mantel-Cox) analysis showed χ^2^ = 53.1, *p* < 0.001, 95% CI (Figure 3B).

### 3.3 Biomarker ROC curves in IPA patients

**Figure 4:**
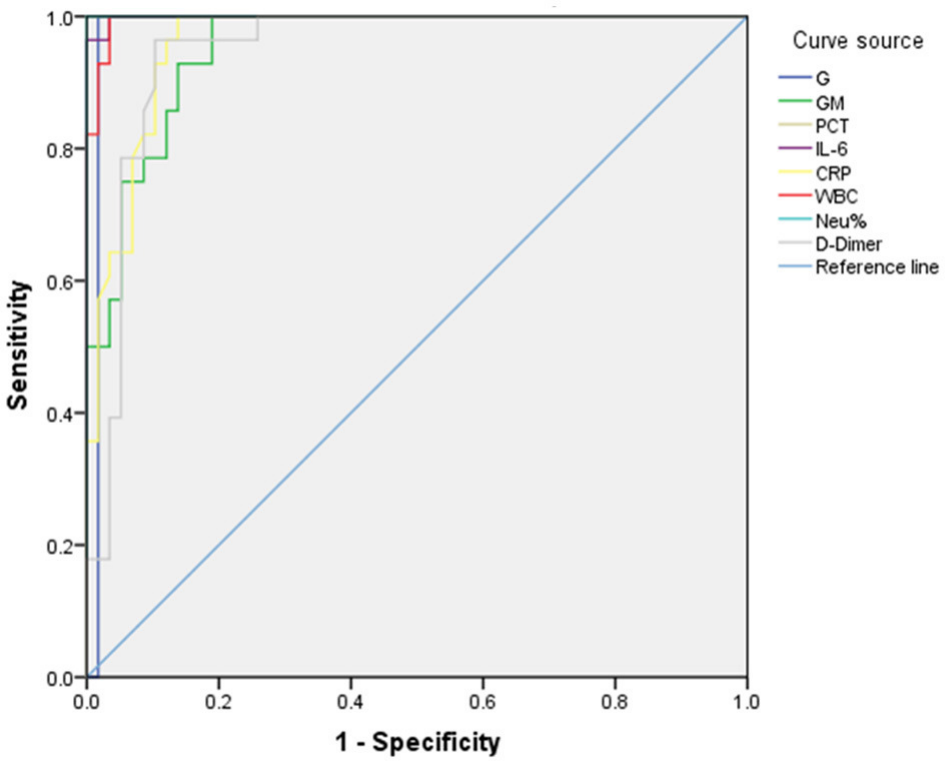
ROC curve for biomarkers in IPA patients. The diagnostic value of biomarkers for IPA was assessed using ROC-AUC analysis. The AUC values for G, GM, PCT, IL-6, WBC, NEU%, and D-Dimer were 0.983, 0.953, 1.000, 0.999, 0.961, 0.996, and 1.000, respectively. With respect to diagnostic thresholds, when G ≥ 131.175 pg/ml, the sensitivity and specificity were 1.000 and 0.948. Similarly, at a GM ≥ 0.510 pg/ml, the sensitivity and specificity were 1.000 and 0.820. When the PCT was ≥ 0.948 ng/ml, the sensitivity and specificity were 1.000 and 0.879. When the WBC was ≥ 16.130 × 109/L, the sensitivity and specificity were 1.000 and 0.966. When the NEU% was ≥ 87.250%, the sensitivity and specificity were 1.000 and 1.000.

The diagnostic value of biomarkers for IPA was assessed using ROC-AUC analysis. The AUC values for G, GM, PCT, IL-6, WBC, NEU%, and D-Dimer were 0.983, 0.953, 1.000, 0.999, 0.961, 0.996, and 1.000, respectively. Concerning diagnostic thresholds, when G ≥ 131.175 pg/ml, the sensitivity and specificity were 1.000 and 0.948. Similarly, at a GM ≥ 0.510 pg/ml, the sensitivity and specificity were 1.000 and 0.820. When the PCT was ≥ 0.948 ng/ml, the sensitivity and specificity were 1.000 and 0.879. When the WBC was ≥ 16.130 × 10^9^/L, the sensitivity and specificity were 1.000 and 0.966. When the NEU% was ≥ 87.250%, the sensitivity and specificity were 1.000 and 1.000 (Figure 4, Table 2).These results are illustrated in Figure 4 and summarized in Table 2. Among 216 IPA patients, 71 patients with serum GM positivity underwent microbial culture, with only 33 (46.5%) yielding positive results. Similarly, among 51 patients with positive culture results, only 27 (52.9%) had GM levels >0.5 pg/ml. Biomarker comparisons between IPA (216 cases) and CPA (72 cases) using the Mann-Whitney U test revealed significant differences in NEU% [*Z* = 3.102, *p* = 0.002, 89.0 (82.2–94.1) vs. 82.1 (74.6–90.2)] and IL-6 [*Z* = 3.712, *p* < 0.001, 57.0 (19.8–228.1) vs. 23.5 (7.3–65.7)].

**Table 2:** The diagnostic value of biomarkers for IPA.

**Biomarkers**	**AUC value**	**Cut-off value**	**Sensitivity**	**Specificity**	**95% Confidence interval**	
					**Lower**	**Upper**
G (pg/ml)	0.983	131.175	1.000	0.948	0.949	1.000
GM (pg/ml)	0.953	0.51	1.000	0.820	0.915	0.992
PCT (ng/ml)	1.000	0.948	1.000	0.879	1.000	1.000
IL-6 (ng/ml)	0.999	121.595	1.000	0.879	0.995	1.000
CRP (mg/ml)	0.961	3.645	1.000	0.845	0.926	0.997
WBC ( × 10^9^/L)	0.996	16.13	1.000	0.966	0.988	1.000
NEU% (%)	1.000	87.25	1.000	1.000	1.000	1.000
D-Dimer (mg/L)	0.946	1.22	1.000	0.741	0.898	0.994

### 3.4 Risk factors associated with IP patient in-hospital death

**Figure 5:**
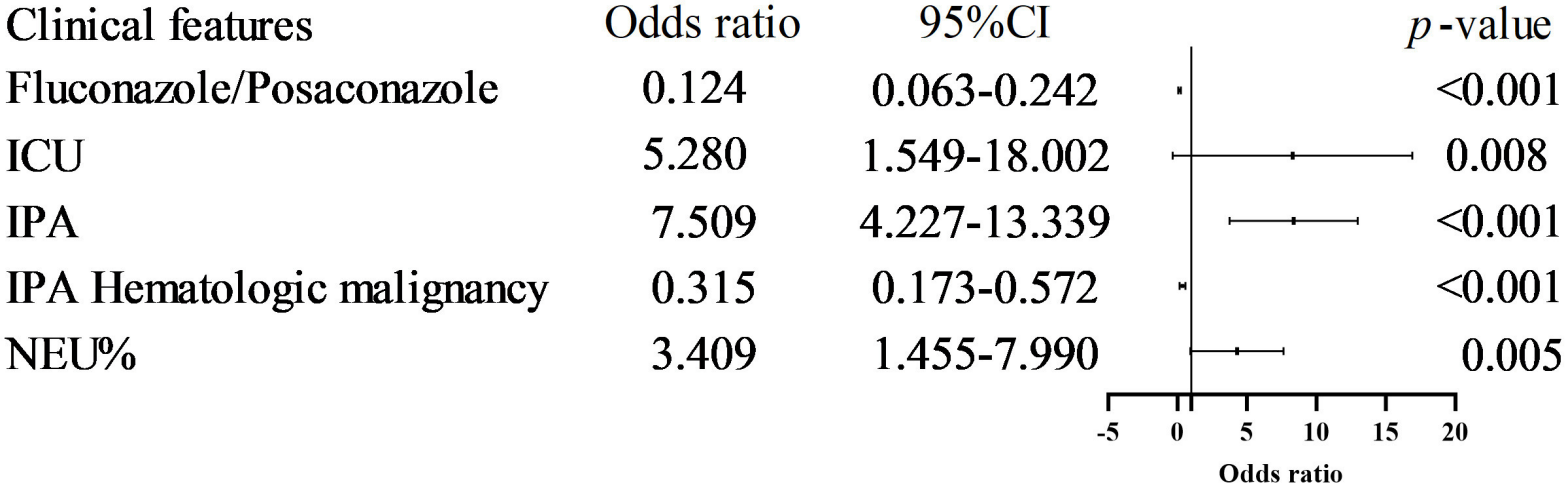
Risk factors associated with IP patient in-hospital death. Multivariate regression analysis revealed that IPA (OR 7.509, 95% CI 4.227–13.339, p < 0.001), Voriconazole/Posaconazole (OR 0.124, 95% CI 0.063–0.242, *p* < 0.001), ICU hospitalization (OR 5.280, 95% CI 1.549-18.002, *p* = 0.008), hematologic malignancy (OR 0.316, 95% CI 0.174-0.573, *p* < 0.001), and NEU% ≥ 87.25% (OR 3.409, 95% CI 1.455–7.990, *p* = 0.005) were associated with in-hospital mortality risk.

Univariate analysis identified several factors associated with in-hospital mortality, including Voriconazole/Posaconazole use, IPA diagnosis, ICU hospitalization, hematologic malignancies, PCT, IL-6, and NEU%. Multivariate regression analysis revealed that IPA (OR 7.509, 95% CI 4.227–13.339, *p* < 0.001), Voriconazole/Posaconazole (OR 0.124, 95% CI 0.063–0.242, *p* < 0.001), ICU hospitalization (OR 5.280, 95% CI 1.549–18.002, *p* = 0.008), hematologic malignancy (OR 0.316, 95% CI 0.174–0.573, *p* < 0.001), and NEU%≥87.25% (OR 3.409, 95% CI 1.455–7.990, *p* = 0.005) were associated with in-hospital mortality risk (Figure 5, Table 3).

**Table 3:** Demographic, clinical and laboratory, findings of patients on admission.

**Demographics and clinical characteristics**	**Total (*n* = 216 cases)**	**Non-survivor**	**Survivor**	***P***
		**(*n* = 148 cases)**	**(*n* = 68 cases)**	
**Sex**				*p*=0.843
Female	90 (42%)	61 (68%)	53 (32%)	
male	126 (58%)	87 (77%)	39 (33%)	
**Age**				*p*=0.446
>=60	112 (52%)	86 (77%)	26 (33%)	
< 60	104 (48%)	62 (60%)	42 (40%)	
**Fluconazole/posaconazole**				*p* < 0.001
Yes	98 (45%)	45 (46%)	53 (54%)	
No	118 (55%)	103 (87%)	15 (13%)	
**Imaging features positive**				*p*=0.450
Yes	103 (48%)	67 (65%)	36 (33%)	
No or not checked	113 (52%)	80 (71%)	33 (39%)	
***Aspergillus*** **disease**				*p* < 0.001
IPA	216 (71%)	148 (69%)	68 (31%)	
CPA	72 (24%)	18 (25%)	54 (75%)	
ABPA	17 (5%)	2 (12%)	15 (88%)	
**ICU hospitalization**				*p* = 0.008
ICU	32 (15%)	29 (91%)	3 (7%)	
Non-ICU	184 (85%)	119 (65%)	65 (35%)	
**GM Value(pg/ml)**				*p* = 0.37
≥0.5 pg/ml	95 (71%)	71 (75%)	20 (25%)	
< 0.5 pg/ml	24 (29%)	17 (71%)	7 (29%)	
**IPA-COVID-19 or H1N1**				*p* = 0.257
Yes	39 (19%)	29 (74%)	10 (24%)	
No	177 (81%)	119 (67%)	58 (33%)	
**IPA Hematologic malignancya**				*p* < 0.001
Yes	86 (40%)	46 (53%)	40 (47%)	
No	130 (60%)	102 (69%)	28 (31%)	
***G*** **value**				*p* = 0.277
≥131 pg/ml	37 (35%)	30 (81%)	7 (19%)	
< 131 pg/ml	70 (65%)	50 (71%)	20 (29%)	
**Pathogenic evidence** ≥**2 times**				*p* = 0.163
No	93 (43%)	59 (63%)	34 (37%)	
Yes	123 (57%)	89 (72%)	34 (28%)	
PCT (ng/ml)	179 [0.38 (0.14, 1.68)]	128 [0.51 (0.19, 2.35)]	51 [0.15 (0.08, 0.93)]	Z = 4.123, *p* < 0.001
IL-6 (ng/ml)	164 [55.4 (18.7, 205.7)]	116 [78.6 (22.8, 228.4)]	48 [26.8 (6.1, 70.3)]	Z = 3.415, *p* < 0.001
G (pg/ml)	107 [60.0 (10.0, 184.0)]	80 [10.5 (61.6, 208.7)]	27 [60.0 (10.0, 158.0)]	Z = 1.409, *p* = 0.159
NEU% (%)	123 [88.8 (82.0, 94.0)]	92 [90.2 (84.7, 94.1)]	31 [82.8 (71.8, 89.0)]	Z = 2.633, *p* = 0.008
WBC( × 10^9^/L)	129 [9.2 (5.6, 16.2)]	95 [9.4 (5.6, 16.3)]	34 [8.4 (4.3, 14.4)]	Z = 0.242, *p* = 0.803
GM(pg/ml)	119 [1.06 (0.58, 3.84)]	89 [1.09 (0.59, 3.87)]	30 [0.73 (0.51, 2.87)]	Z = 1.279, *p* = 0.201
D-dimer (mg/L)	29 [6.2 (3.2, 12.6)]	23 [6.2 (3.3, 12.9)]	6 [3.7 (1.3, 12.4)]	Z = 0.431, *p* = 0.667
CRP (mg/L)	39 [9.9 (4.3, 12.9)]	29 [10.2 (6.9, 14.9)]	10 [5.4 (2.2, 10.5)]	Z = 2.171, *p* = 0.030

Data are median (IQR) or n (%). *p*-values were calculated by Mann-Whitney U test, χ2 test, or Fisher's exact test, as appropriate.

### 3.5 IPA CT manifestations

A total of 121 patients diagnosed with invasive pulmonary aspergillosis (IPA) underwent CT imaging. Among these, 104 patients (85.9%) exhibited abnormal imaging findings. Of these 104 patients, 73 (60.3%) demonstrated imaging features consistent with fungal infections. The specific imaging manifestations Patchy shadow (34 cases,28.1%) was the most predominant finding, followed by Ground-glass opacities at 12 cases (9.9%), Mass-like shadows at 11 cases (9.1%), Consolidation at 7 cases (5.8%), Cavitary lesions at 5 cases (4.1%), Nodular shadows at 2 cases (1.6%), and Aspergilloma at 2 cases (1.6%).

## 4 Discussion

Invasive pulmonary aspergillosis (IPA) is a leading cause of morbidity and mortality in immunocompromised patients, and its diagnosis remains challenging (Gaffney et al., 2023). In this study, IPA accounted for 62.6% of all pulmonary aspergillosis cases, higher than in prior reports (Gaffney et al., 2023) that in COVID-19-IPA patients IAPA accounted for 54.0%. The fungal culture results revealed that *A. fumigatus* was the primary pathogenic microorganism, accounting for 61.5% of cases, when analyzing BALF and qualified sputum samples. Consistent with previous reports (Rudramurthy et al., 2019), A. fumigatus was identified as the leading cause of pulmonary aspergillosis. However, quantitative cultures from lower respiratory specimens cannot reliably distinguish infection from colonization. Therefore, additional evidence—such as compatible host factors, imaging findings, and biomarkers like GM and G tests—is crucial to support an IPA diagnosis (Jenks et al., 2021). In this study, the host factors associated with IPA included hematologic malignancy (33.8%), ICU admission (18.5%), COVID-19-associated IPA (16.2%), cancers (13.9%), malignant tumors (2.3%), and non-immunocompromised hosts (2.3%). *Aspergillus* is a major cause of infectious mortality among severely immunocompromised patients (Latgé and Chamilos, 2019), ICU patients (Rudramurthy et al., 2019), those with COVID-19-associated pulmonary aspergillosis (Koehler et al., 2021), and non-immunocompromised patients (Tudesq et al., 2019), highlighting the susceptibility of a broad patient population.

The mortality rate of IPA patients in this study was 68.5%, exceeding the >50% mortality rate reported in the literature for severely immunocompromised and ICU patients (Gaffney et al., 2023; Jenks et al., 2021). The use of Voriconazole/Posaconazole in our cohort was 45% (98/216) overall. Among patients with hematologic malignancies, the usage rate was 70% (66/95), and their 90-day survival rate was 0.538 ± 0.055, which is higher than the literature-reported 75.2% mortality rate in hematologic malignancy-associated IPA diagnosed before ICU admission (Pardo et al., 2019). IPA In contrast, non-hematologic malignancy patients had a much lower usage rate (26%, 32/121) and a 90-day survival rate of only 0.106 ± 0.034. This disparity may stem from current guidelines emphasizing IPA's prevalence in hematologic malignancies (Latgé and Chamilos, 2019; Patterson et al., 2016; Donnelly et al., 2020; Hage et al., 2019; Ullmann et al., 2018), leading to delayed diagnosis or missed antifungal treatment in non-hematologic malignancy patients. This finding is among the most significant in this study. The median survival time for patients treated with Voriconazole/Posaconazole was 20 days (95% CI, 15.8–24.2) vs. 130 days for those without treatment (χ^2^ = 53.1, *p* < 0.001). Similarly, the 42-day survival rate with Voriconazole/Posaconazole was 0.674 ± 0.052 compared to 0.227 ± 0.040 without treatment. These rates are lower than the 42-day mortality reported in phase 3 trials, where mortality was 15% for posaconazole, 21% for voriconazole (Maertens et al., 2021), and the 42-day all-cause mortality for isavuconazole was 18% as compared to 20% for voriconazole (Maertens et al., 2016).

Non-invasive biomarkers can enable the early diagnosis of IPA, including the GM and G tests (Latgé and Chamilos, 2019). In our study, the diagnostic value (AUC) for serum GM was 0.953, with a sensitivity and specificity of 1.000 and 0.820 (95% CI) at a cutoff of GM ≥ 0.510 pg/ml. These results are higher than literature-reported sensitivity and specificity values of 74% and 85%, respectively, at a 0.5 pg/ml cutoff (Jenks et al., 2021) but similar to the findings of Bukkems et al. (2023), who measured sensitivity and specificity values of 0.92 and 0.84, respectively, at a serum GM cutoff of 0.5 pg/ml, potentially owing to differences in reagent quality. Among 216 IPA patients, 71 patients with serum GM positivity underwent microbial culture, with only 33 (46.5%) yielding positive results. Similarly, among 51 patients with positive culture results, only 27 (52.9%) had GM levels >0.5 pg/ml, due to low sensitivity of GM in blood (Jenks et al., 2021), potentially explaining the low positive predictive value of microbial culture and GM for IPA. The AUC value when assessing the diagnostic performance of G for IPA was 0.983 at a cutoff of G≥131.175 pg/ml, with respective sensitivity and specificity values of 1.000 and 0.948. This is consistent with results from Springer et al. (2021) using a cutoff of 306 pg/mL, as they achieved 90% specificity in the acute posttransplant phase. The AUC for PCT in diagnosing IPA was 1.000, with sensitivity and specificity of 1.000 and 0.879 (95% CI) at PCT ≥ 0.948 ng/ml, which is inconsistent with the results from Springer et al. (2021), who failed to detect any significant utility for PCT when diagnosing IPA in COPD patients. For NEU%, the AUC was 1.000, and a cutoff of NEU% ≥ 87.250% yielded sensitivity and specificity values of 1.000 (95% CI). This finding has not been previously reported. While biomarkers like G, PCT, and NEU% are useful for other infections, no single test offers high predictive value for IPA. Therefore, a comprehensive diagnosis incorporating biomarkers and mycological evidence is essential.

Univariate analysis identified Voriconazole/Posaconazole (OR 0.124, 95% CI 0.063–0.242, *p* < 0.001) and hematologic malignancy (OR 0.316, 95% CI 0.174–0.573, *p* < 0.001) as protective factors associated with in-hospital mortality. Voriconazole/Posaconazole significantly reduced overall IPA-related mortality (Maertens et al., 2021, 2016; Hoenigl et al., 2018). Conversely, IPA was strongly associated with in-hospital death (odds ratio 7.509, 95% CI 4.227–13.339, *p* < 0.001), consistent with results published by Cornely et al. (2009). ICU hospitalization was another risk factor identified through this approach (OR 5.280, 95% CI 1.549–18.002, *p* = 0.008), aligning with reports of increased short- and long-term mortality in ICU IPA patients (Delsuc et al., 2015). Other risk factors for in-hospital mortality included NEU% ≥ 87.25% (OR 3.409, 95% CI 1.455–7.990, *p* = 0.005) and biomarkers such as NEU% as an IPA infection biomarker, in non-neutropenic patients, a high NEU% may indicate a more severe infection or inflammatory response. PCT and IL-6 although corresponding literature reports are lacking.

Chest CT is an indispensable tool for diagnosing *Aspergillus* infections, particularly in high-risk populations like immunocompromised patients. In our study, 121 IPA patients 60.3% demonstrated imaging features consistent with fungal infections, lower than Wu and Huang (2020) reported the sensitivity of CT in the diagnosis of IPA was 75.0% in hematologic patients. T The specific imaging manifestations Patchy shadow (34 cases, 28.1%) was the most predominant finding, followed by Ground-glass opacities at 12 cases (9.9%) and Mass-like shadows at 11 cases (9.1%), multiple patchy shadows in both lungs were the most common CT signs of suspected and confirmed patients (Wu and Huang, 2020; Tian et al., 2021).

Given the high mortality and low diagnostic rates of IPA, incorporating biomarkers, CT, and mycological evidence into comprehensive diagnostic approaches is crucial. Effective use of Voriconazole/Posaconazole remains essential as a means of reducing in-hospital mortality among IPA patients.

## Data Availability

The original contributions presented in the study are included in the article/supplementary material, further inquiries can be directed to the corresponding author.
